# ANGPTL4 promotes the progression of cutaneous melanoma to brain metastasis

**DOI:** 10.18632/oncotarget.19018

**Published:** 2017-07-05

**Authors:** Sivan Izraely, Shlomit Ben-Menachem, Orit Sagi-Assif, Tsipi Meshel, Diego M. Marzese, Shuichi Ohe, Inna Zubrilov, Metsada Pasmanik-Chor, Dave S.B. Hoon, Isaac P. Witz

**Affiliations:** ^1^ Department of Cell Research and Immunology, George S. Wise Faculty of Life Sciences, Tel Aviv University, Tel Aviv, Israel; ^2^ Department of Translational Molecular Medicine, John Wayne Cancer Institute at Providence Saint John's Health Center, Santa Monica, CA, USA; ^3^ Bioinformatics Unit, The George S. Wise Faculty of Life Sciences, Tel Aviv University, Tel Aviv, Israel

**Keywords:** ANGPTL4, melanoma, brain, metastasis, TGFβ1

## Abstract

In an ongoing effort to identify molecular determinants regulating melanoma brain metastasis, we previously identified Angiopoietin-like 4 (ANGPTL4) as a component of the molecular signature of such metastases.

The aim of this study was to determine the functional significance of ANGPTL4 in the shaping of melanoma malignancy phenotype, especially in the establishment of brain metastasis.

We confirmed that ANGPTL4 expression is significantly higher in cells metastasizing to the brain than in cells from the cutaneous (local) tumor from the same melanoma in a nude mouse xenograft model, and also in paired clinical specimens of melanoma metastases than in primary melanomas from the same patients.

In vitro experiments indicated that brain-derived soluble factors and transforming growth factor β1 (TGFβ1) up-regulated ANGPTL4 expression by melanoma cells.

Forced over-expression of ANGPTL4 in cutaneous melanoma cells promoted their ability to adhere and transmigrate brain endothelial cells. Over-expressing ANGPTL4 in cells derived from brain metastases resulted in the opposite effects.

In vivo data indicated that forced overexpression of ANGPTL4 promoted the tumorigenicity of cutaneous melanoma cells but did not increase their ability to form brain metastasis. This finding can be explained by inhibitory activities of brain-derived soluble factors.

Taken together these findings indicate that ANGPTL4 promotes the malignancy phenotype of primary melanomas of risk to metastasize to the brain.

## INTRODUCTION

Malignant melanoma has a high tendency to develop brain metastasis [1], conferring upon melanoma patients a very poor prognosis [2]. As disease management improves and the survival of melanoma patients is prolonged, the number of patients that eventually develop clinical brain metastasis increases. Treatment options are limited beyond surgery [3] and survival of treated patients depends on tumor size, metastasis location, and number of tumor metastasis [3].

The brain microenvironment consists of several resident cell types: endothelial cells, astrocytes, microglia, and neurons. To develop brain metastasis, melanoma cells must reach the brain vasculature, attach to endothelial cells covering the microvessel walls, extravasate the brain endothelium and invade the brain parenchyma, proliferate and induce angiogenesis [4]. Interactions with brain microenvironmental cells facilitate metastasis formation. Astrocytes, for example, interact with tumor cells in the brain whereby, these interactions may promote metastasis growth [5, 6]. Furthermore, interaction of glioma initiating cells with macrophages/microglia up-regulate B7-H4 expression in the latter cells, blocking effective T-cell immune responses [7]. The molecules and mechanisms involved in the interactions between melanoma cells and normal brain microenvironment cells have not been fully characterized.

We developed human melanoma xenograft models encompassing cutaneous (local tumor) and melanoma brain metastasis (MBM) variants originating from single melanoma tumors [8]. These models have 2 major tumor biologic relevancies: 1. Both types of variants share a common genetic background. 2. MBM cells originate from cells exposed initially to the microenvironment of the primary tumor and subsequently to the metastatic microenvironment of the brain while the cutaneous variants experienced the former microenvironment. This model enables thus to dissect melanoma characteristics that were influenced by the microenvironment of the local cutaneous tumor from those conferred by the metastatic microenvironment.

Previous experiments using these models indicated that several genes such as *ANGPTL4, CCR4, PTGS2, MMP1,* and *PRAME* are more highly expressed by human MBM cells than by the respective cutaneous variants. Other genes such as *ITGA4, CLDN1, CYR61,* *CDH1,* and *IL6R* are aberrantly down-regulated in brain metastases [8, 9]. Our functional studies indicated that claudin-1 (CLDN1) is a MBM suppressor [10] and recently that CCR4 is a MBM promoter [11].

Angiopoietin-like 4 (ANGPTL4) is a secreted cytokine member of the angiopoietin family of vascular regulators [12]. Angiopoietin-like proteins take part in endothelial cell survival, adhesion and paradoxically, stimulation or inhibition of angiogenesis and vascular leakiness [12, 13].

ANGPTL4 acts as a tumor suppressor or promoter of cancer metastasis, depending on cell type and stage of cancer [14]. ANGPTL4 regulates diverse malignant processes. It disrupts vascular endothelial cell-cell tight junctions (TJ) and adherence junctions, facilitates trans-endothelial passage of tumor cells, regulates cell proliferation, apoptosis, angiogenesis, adhesion, motility and wound healing and acts as an immunosuppressive factor [12, 15]. ANGPTL4 is also correlated with brain metastasis relapse in breast cancer [16]. However, some studies demonstrated the opposite effects [17].

A further investigation is needed using our brain metastasis model to better understand how the tumor microenvironment influences the function of ANGPTL4 in early stages of MBM.

## RESULTS

### Brain metastasizing melanoma variants over-express ANGPTL4

**Figure 1:**
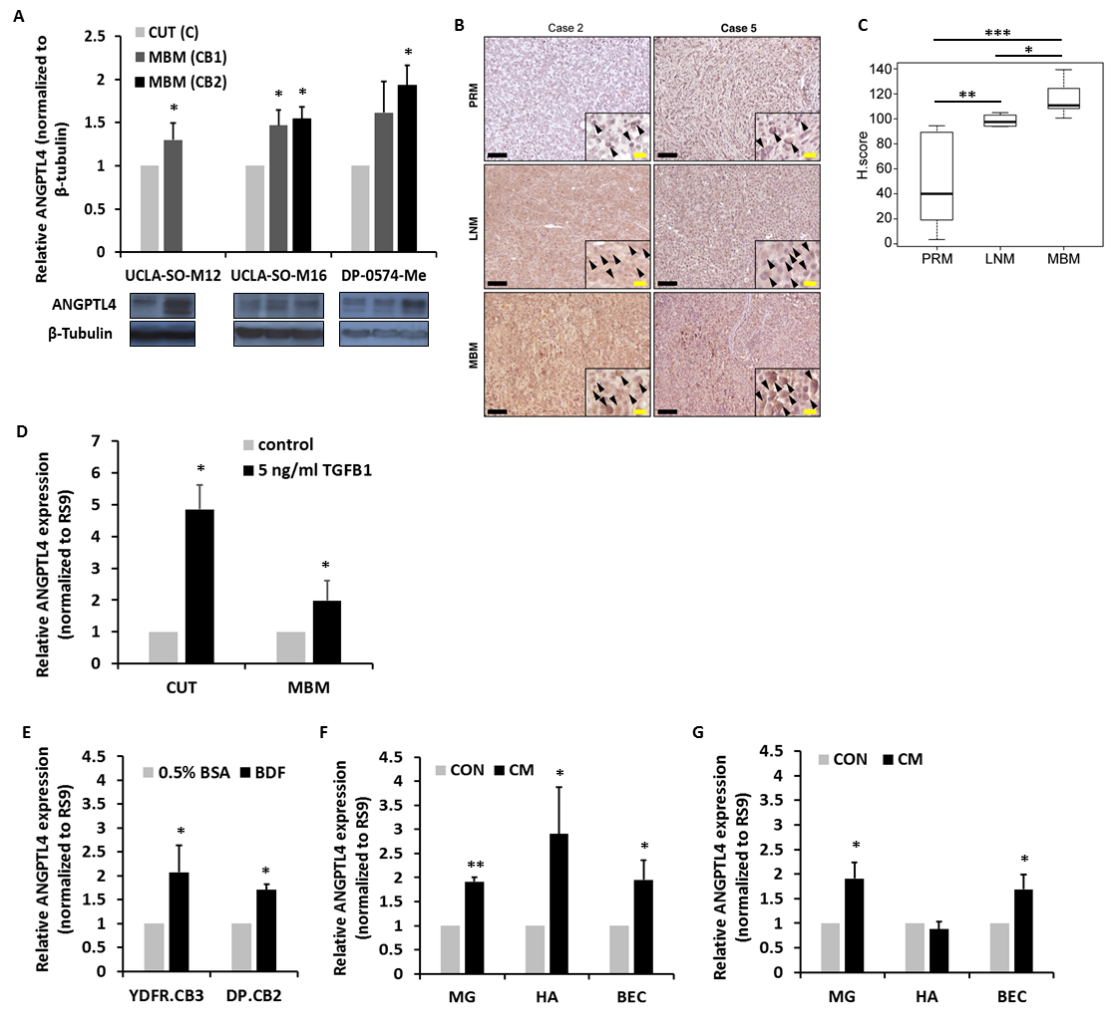
ANGPTL4 expression during melanoma progression to brain metastasis **A.** ANGPTL4 protein expression level in UCLA-SO-M12, UCLA-SO-M16 and DP-0574-Me cutaneous (CUT) and melanoma brain metastasizing (MBM) variants of first and second IC inoculation cycle was analyzed using Western blotting. The obtained values were normalized to β-Tubulin. The bars represent the relative expression of ANGPTL4 (normalized to RS9), compared to control, untreated cells + SD obtained in one measurement in at least three independent experiments. *P < 0.05. **B.**, **C.** ANGPTL4 expression in paired samples of primary melanoma (PRM), melanoma lymph node metastasis (LNM), and melanoma brain metastasis (MBM) derived from melanoma patients. (B) Representative IHC staining with anti-ANGPTL4 Ab for PRM, LNM and MBM specimens. Black bars indicate 100µm. The insets show a magnification of the melanoma lesions. Black arrowheads indicate ANGPTL4 positive melanoma cells. Yellow bars indicate 20µm. (C) Box plot comparing H score for PRM, LNM and MBM. *P < 0.01, **P < 0.005, ***P < 0.0005. **D.** Melanoma cells were incubated with 5ng/ml TGFβ1 for 4 hrs. Following stimulation, RT-qPCR analysis was performed to determine the mRNA expression level of ANGPTL4. The bars represent the relative expression of ANGPTL4 (normalized to RS9), compared to control, untreated cells + SD obtained in one measurement in at least three independent experiments. *P < 0.05. **E.** Brains of BALB/c mice were harvested, and BDF were prepared after 24 hrs (see Materials and Methods) and added to melanoma cells for 24 hrs at 37°C. Melanoma cells treated with 0.5% BSA supplemented RPMI-1640 served as control. Following stimulation, RT-qPCR analysis was performed to determine the mRNA expression level of ANGPTL4. The bars represent the relative expression of ANGPTL4 (normalized to RS9), compared to control, untreated cells + SEM obtained in one measurement in at least three independent experiments. *P < 0.05. **F.**, **G.** CM of microglia (MG), astrocytes (HA) and BEC was collected, and added to YDFR.CB3 (F) and DP.CB2 (G) melanoma cells for 24 hrs at 37°C. Melanoma cells treated with starvation medium served as control. Following stimulation, RT-qPCR analysis was performed to determine the mRNA expression level of ANGPTL4. The bars represent the relative expression of ANGPTL4 (normalized to RS9), compared to control, untreated cells + SEM obtained in one measurement in at least three independent experiments. *P < 0.05, **P < 0.005.

In a previous study we showed that MBM variants of 3 different human melanoma xenograft models express higher levels of ANGPTL4 than their corresponding cutaneous variants [8]. These findings were confirmed in three additional independent melanoma models: by using Western blot analysis, we assessed ANGPTL4 expression in cutaneous and MBM cells of the parental human melanoma cells UCLA-SO-M12, UCLA-SO-M16, and DP-0574-Me. A significant higher expression of ANGPTL4 was observed in the brain macro-metastatic variants of these melanomas than in the corresponding cutaneous variants (*P* < 0.05) (Figure 1A). Remarkably, we also identified that ANGPTL4 is up-regulated in MBM clinical samples. The expression of ANGPTL4 was measured in a cohort of 12 melanoma patients with paired primary melanoma (PRM), melanoma lymph node metastasis (LNM), and MBM. Autologous paired triplets (PRM; LNM; MBM) were derived from 8 patients, paired duplets (PRM-LNM) or (LNM-MBM) were derived from 3 patients and a single MBM was derived from one patient. Immunohistochemistry (IHC) staining indicated that LNM and MBM exhibited significantly higher expression of ANGPTL4 (*P* < 0.005 and *P* < 0.0005, respectively) than paired PRM, and that MBM exhibited significantly (*P* < 0.01) higher expression of ANGPTL4 than paired LNM (Figure 1B, 1C).

### ANGPTL4 expression is up-regulated by tumor growth factor β1 (TGFβ1)

Melanoma cells can secrete high amounts of TGFβ1, inducing its own expression through a positive feedback loop [18]. Elevated TGFβ1 plasma levels have been shown to correlate with melanoma metastatic progression whereby, this cytokine is known to promote tumor progression [19]. The TGFβ1 signaling axis regulates the expression of a variety of genes such as cytokines, angiogenic factors, extracellular matrix (ECM) components and cell-surface receptors [12].

We tested whether TGFβ1 regulates ANGPTL4 in cutaneous and MBM cells. Melanoma cells were stimulated with 5ng/ml TGFβ1 for 4 hrs. Respective control cells remained untreated. Reverse transcription quantitative real-time PCR (RT-qPCR) analysis was performed to determine ANGPTL4 mRNA expression level. The results showed that TGFβ1 significantly up-regulated ANGPTL4 expression in both the cutaneous and MBM cells (*P* < 0.05) (Figure 1D). ANGPTL4 up-regulation in cutaneous and MBM cells was confirmed at the protein level after a 24 hrs treatment (in a fold change of 3.16 and 1.34, respectively) and after a 48 hrs treatment (in a fold change of 3.59 and 2.8, respectively) with 5ng/ml TGFβ1, compared to control, untreated cells.

### ANGPTL4 expression is up-regulated by brain-derived factors (BDF)

In order to test whether the brain microenvironment modulates the expression of ANGPTL4 in melanoma cells, melanoma cells were subjected to BDF (see Materials and Methods) for 24 hrs, and then tested for ANGPTL4 expression. In Figure 1E we demonstrated that BDF significantly up-regulated the expression of ANGPTL4, compared to its expression in respective control cells (*P* < 0.05). ANGPTL4 up-regulation in YDFR.CB3 and DP.CB2 cells was confirmed at the protein level after a 48 hrs treatment with BDF (in a fold change of 1.5 and 1.46, respectively), compared to control, untreated cells. In order to examine which cells of the normal brain environment are the source of the soluble products that up-regulate ANGPTL4 expression, we performed similar experiments with conditioned medium (CM) of microglia, astrocytes, and brain endothelial cells (BEC). Figure 1F showed that soluble factors of these three cells increased ANGPTL4 expression in YDFR.CB3 cells (*P* < 0.05). In addition, in Figure 1G it was demonstrated that soluble factors of microglia and BEC increased ANGPTL4 expression in DP.CB2 melanoma cells (*P* < 0.05).

### Overexpression or knockdown of ANGPTL4 in melanoma cells

**Figure 2:**
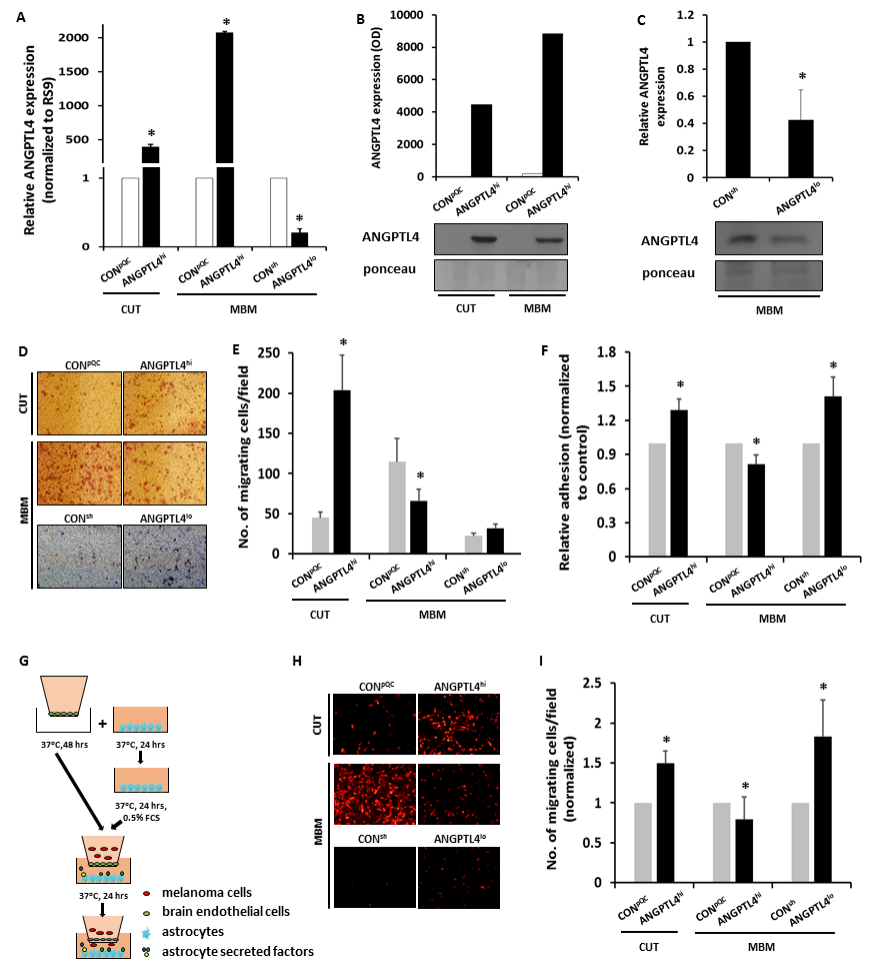
ANGPTL4 controls the malignancy phenotype of cutaneous and brain metastasizing melanoma variants **A.**-**C.** Cutaneous (CUT) and melanoma brain metastasizing (MBM) variants were transduced with an ANGPTL4 cDNA-containing construct (ANGPTL4^hi^) or with the backbone construct pQCXIP (CON^pQC^). MBM cells were transduced with a mixture of 4 different shANGPTL4-containing constructs (ANGPTL4^lo^), or with the control construct (CON^sh^). The efficacy of ANGPTL4 over-expression or down-regulation was verified: (A) RT-qPCR analysis was performed to determine the mRNA expression level of ANGPTL4. The bars represent the relative ANGPTL4 expression (normalized to RS9) in ANGPTL4^hi^ or ANGPTL4^lo^ cells compared to control cells + SEM obtained in one measurement in at least three independent experiments. *P < 0.05. (B, C) Supernatants of the transduced cells were subjected to Western blot analysis. ANGPTL4 was detected by specific Abs. The bars represent the relative ANGPTL4 expression in ANGPTL4^hi^ or ANGPTL4^lo^ cells compared to control cells + SEM obtained in one measurement in two-three independent experiments. *P < 0.05. Representative blots are shown. Ponceau staining was used for loading control. **D.**, **E.** Melanoma cells were allowed to migrate through collagen coated transwells for 24 hrs. The migrated cells were fixed and counted. (D) Representative images are presented (X10 magnification). (E) The bars represent the average number of migrating cells per field in three independent experiments performed in triplicates + SEM. *P < 0.05. **F.** BEC were cultured for 24 hrs to form a confluent monolayer and stimulated with 100 units/ml TNFα and IFNγ for additional 24 hrs. mCherry- or GFP-expressing melanoma cells were added and allowed to adhere for 30 min at 37°C. The fluorescence signal of labeled cells was measured before and after removal of non-adherent cells. The bars represent the average % adherent cells (normalized to control cells) + SEM in at least three independent experiments. Six replicates were performed in each experiment. *P < 0.05. **G.**-**I.** BEC were cultured for 48 hrs on the upper side of the apical chamber of transwell inserts to form a confluent monolayer. Astrocytes were cultured in 24-well plates for 24 hrs and starved for additional 24 hrs to allow the secretion of soluble factors. mCherry-expressing melanoma cells were added onto the BEC monolayer and allowed to migrate for 24 hrs towards the astrocytes (G). The migrated cells were fixed and counted. (H) Representative images are presented (X10 magnification). (I) The bars represent the average number of migrating cells per field (normalized to control cells) in at least three independent experiments performed in triplicates + SEM. *P < 0.05.

Cutaneous and MBM cells were infected with virions composed of constructs containing ANGPTL4 cDNA, to establish cell populations over-expressing ANGPTL4 (ANGPTL4^hi^ cells). The same cells, infected with virions containing a mock plasmid, were used as control (CON^pQC^). Over-expression of ANGPTL4 was validated using both RT-qPCR and Western blotting (Figure 2A, 2B). To down-regulate endogenous ANGPTL4 expression, we infected MBM cells with virions containing a mixture of four different shANGPTL4-pGIPZ plasmids (ANGPTL4^lo^).

Using RT-qPCR and Western blotting we examined these four plasmids for their efficacy to down-regulate the expression of this protein (data not shown). A mixture of the four vectors was used to maximize ANGPTL4 knock down as it showed the strongest effect (Figure 2A, 2C).

The same cells, infected with virions containing sh-non-silencing pGIPZ vector, were used as control (CON^sh^).

### ANGPTL4 regulates the migratory capacity of melanoma cells

The invasion of cancer cells into surrounding tissues as well as their intravasation and extravasation requires migration steered by protrusive activity of the cell membrane and its attachment to the ECM [20]. Since ANGPTL4 alters the migratory capacity of several cell types [13, 21], we examined if ANGPTL4 over-expressing melanoma cells would manifest an altered ability to migrate through collagen coated transwells. Cutaneous ANGPTL4^hi^ cells migrated more efficiently than the corresponding CON^pQC^ cells (*P* < 0.05) (Figure 2D, 2E). In contrast, ANGPTL4^hi^ MBM cells migrated less efficiently than the corresponding CON^pQC^ cells (*P* < 0.05).

We also examined if ANGPTL4 down-regulation alters the ability of melanoma cells to migrate through collagen coated transwells. The results indicated that ANGPTL4 knock down in MBM cells does not affect these tumor cells migratory and invasion ability (Figure 2D, 2E).

### ANGPTL4 regulates the adhesion of melanoma cells to BEC

In order to disseminate the brain, melanoma cells must penetrate the brain endothelium. An initial step in this process is the adhesion of cancer cells to the endothelium [22]. We therfore evaluated changes in the capacity of cutaneous and MBM cells over-expressing ANGPTL4 to adhere to BEC. ANGPTL4^hi^ cutaneous cells adhered better to BEC than the controls. On the other hand ANGPTL4^hi^ MBM cells adhered less well to BEC than the corresponding CON^pQC^ cells (*P* < 0.05) (Figure 2F). We also determined the adherence capacity of GFP-expressing ANGPTL4^lo^ MBM cells and of CON^sh^ cells to BEC. ANGPTL4^lo^ MBM cells adhered better to BEC (*P* < 0.05) than their corresponding CON^sh^ cells (Figure 2F). Taken together these results indicated that ANGPTL4 is a determinant of the adherence capacity of melanoma cells to BEC. Whereas ANGPTL4 promotes the adhesion of cutaneous cells to BEC it reduces the adhesion of MBM cells to BEC.

### ANGPTL4 regulates melanoma cell transmigration through BEC

In a previous study we demonstrated that astrocytes have the capacity to chemo-attract melanoma cells thereby enhancing their ability to transmigrate through BEC [5]. We therefore evaluated whether ANGPTL4 affecs the transendothelial migration (TEM) of melanoma cells in a blood-brain barrier (BBB) model simulating the extravasation of melanoma cells through BEC towards astrocytes. Figure 2H, 2I showed that ANGPTL4 over-expression significantly enhanced the ability of m-Cherry labeled cutaneous melanoma cells to migrate towards astrocytes through BEC (*P* < 0.05). In contast, ANGPTL4 over-expression attenuated the TEM of MBM cells (*P* < 0.05). MBM cells in whom ANGPTL4 was knocked down (ANGPTL4^lo^ cells) transmigrated more efficiently towards astrocytes through BEC than the corresponding CON^sh^ cells (*P* < 0.05).

The above results indicated that ANGPTL4 is functionally involved in the migration and TEM of melanoma cells. Whereas ANGPTL4 enhanced TEM of cutaneous melanoma cells through the blood brain barrier, it reduced TEM of the MBM cells through the blood brain barrier. The observation that ANGPTL4 influenced differentialy the ability of cutaneous and MBM cells to transmigrate through BEC layers may be due, at least in part, to the differential capacity of the two types of cells to adhere to brain-derived endothelium.

An alternative explanation is that when over-expressed by the MBM cells, ANGPTL4 may increase the stability of endothelial junctions and thus increase endothelial cell barrier integrity, as demonstarted by Bouleti et al [23].

### Secretion of factors that differentially modify the viability of BEC

**Figure 3:**
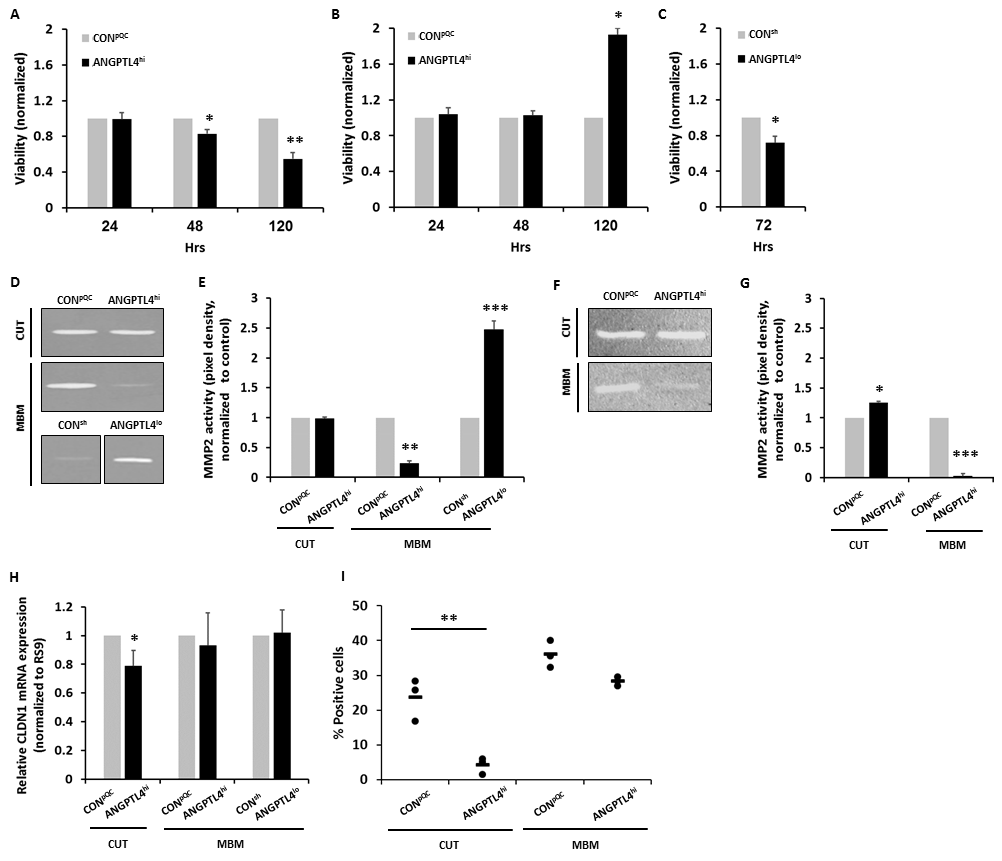
Secretion of bioactive factors from melanoma cells is dependent on ANGPTL4 expression level **A.**-**C.** Melanoma cells were cultured for 24 hrs, then starved for additional 24 hrs, to allow secretion of melanoma-soluble factors. CM was collected and added to BEC. Cell viability of BEC grown with CM of cutaneous CON^pQC^ and ANGPTL4^hi^ melanoma cells (A) or MBM CON^pQC^ and ANGPTL4^hi^ melanoma cells (B) was monitored after 24, 48 and 120 hrs. Cell viability of BEC grown with CM of MBM CON^sh^ and ANGPTL4^lo^ melanoma cells (C) was monitored after 72 hrs. Viability was determined using XTT-based assay. Absorbance at 450 nm was determined for each well, and subtraction of nonspecific readings (measured at 630 nm) was calculated. The bars represent the average viability of the BEC grown with ANGPTL4^hi^ or ANGPTL4^lo^ melanoma CM relative to those grown in control melanoma CM + SD in three independent experiments. Six replicates were performed in each experiment. *P < 0.05, **P < 0.05. **D.**-**G.** Melanoma cells were seeded for 24 hrs and then cultured in serum-free media for additional 24 hrs. CM were collected and subjected to gelatin and collagen zymography. (D, F) Images of one representative experiment out of three are presented. A ~66 kDa band indicating the active form of MMP-2 was observed. (E, G) The bars represent the average MMP-2 activity (pixel density) as measured by densitometry, normalized to control cells + SD in one measurement in three independent experiments. *P < 0.05, **P < 0.0.1, ***P < 0.005. **H.**, **I.** BECs were treated with melanoma CM for 24 hours. Then, CLDN1 expression was analyzed using RT-qPCR and flow cytometry. (H) The bars represent the relative CLDN1 mRNA expression (normalized to RS9) compared to control cells + SD obtained in one measurement in least three independent experiments. *P < 0.05. (I) BEC were trypsinized and analyzed for CLDN1 expression using flow cytometry. Dot plot histogram comparing % CLDN1 positive BEC. Each dot represents the value obtained in a single independent experiment. The line represents the average value in each variant. **P < 0.01.

Endothelial cell status and growth is a crucial factor in angiogenesis [24] whereby the cross-talk between tumor and endothelial cells contributes to these functions. To determine whether ANGPTL4 overexpressing melanoma cells differ from control cells in the ability to sustain the viability of BEC, BEC were treated with CM of CON^pQC^ or ANGPTL4^hi^ cutaneous and MBM cells. XTT assays indicated that CM of ANGPTL4^hi^ cutaneous melanoma cells decreased the viability of BEC (compared to the viability of BEC exposed to CM of CON^pQC^ melanoma cells), at 48 and 120 hrs of exposure (*P* < 0.05 and *P* < 0.005, respectively) (Figure 3A).

On the other hand at 120 hrs of exposure, CM of ANGPTL4^hi^ MBM cells increased the viability of BEC compared to that of BEC exposed to CM of CON^pQC^ cells (*P* < 0.05) (Figure 3B).

Similar experiments were performed with CM of CON^sh^ and ANGPTL4^lo^ MBM cells. At 72 hrs of exposure the viability of BEC exposed to CM of ANGPTL4^lo^ brain metastatic melanoma cells was lower than the viability of BEC exposed to CM of CON^sh^ cells, (*P* < 0.05) (Figure 3C).

Taken together these results demonstrated that ANGPTL4 expressed by cutaneous melanoma cells induces or up-regulates secretion of soluble factors that inhibit BEC growth. In contrast ANGPTL4 in MBM cells induces or up-regulates secretion of soluble factors that promote BEC growth. This indicates the ANGPTL4 expression effect is context dependent on the source of the melanoma cell.

### Secretion of active matrix metalloproteinase-2 (MMP-2)

Invasive growth of cancer cells requires local proteolysis, which is mediated by locally secreted MMPs. These enzymes participate in ECM degradation during metastasis, and are essential for basement-membrane penetration [25]. An increased expression of MMP proteins, such as MMP-2 in tumor cells is often accompanied with increased invasiveness and metastasis, as well as with decrease in overall survival [26].

To determine if ANGPTL4 is involved in the regulation of MMP-2 activity, we performed gelatin and collagen zymography assays comparing cutaneous and MBM cells overexpressing ANGPTL4 (ANGPTL4^hi^) with appropriate control cells (CON^pQC^).

Whereas CON^pQC^ and ANGPTL4^hi^ cutaneous cells secreted higher levels of active collagen degrading MMP-2 (*P* < 0.05) (Figure 3F, 3G), ANGPTL4^hi^ MBM cells secreted significantly lower levels of active gelatin and collagen degrading MMP-2, than CON^pQC^ cells (*P* < 0.01 and P < 0.0005, respectively) (Figure 3D-3G).

We next examined if ANGPTL4 down-regulation in melanoma cells affects MMP-2 secretion. Gelatin zymography assays comparing the CON^sh^ and ANGPTL4^lo^ MBM cells showed that ANGPTL4^lo^ MBM cells secreted higher levels of active gelatin degrading MMP-2 than CON^sh^ cells (*P* < 0.005) (Figure 3D, 3E).

Taken together, these results imply that ANGPTL4 inhibits MMP-2 activity in MBM cells, while it has an inducing effect on MMP-2 activity in the cutaneous cells. This suggests that ANGPTL4 contributes to melanoma cell invasion from the primary tumor to enhance metastatic spread.

### Secretion of factors that differentially decrease expression of CLDN1 by BEC

As ANGPTL4 reprograms endothelial permeability [15, 27] we asked if melanoma-associated ANGPTL4 regulates either directly or indirectly the integrity of TJs in BEC. We utilized RT-qPCR and flow cytometry to measure the expression of CLDN1, a TJ protein known to be expressed on the BEC [10]. BEC were treated with CM of CON^pQC^ or of ANGPTL4^hi^ cutaneous and MBM cells. Figures 3H and 3I show that CM of ANGPTL4^hi^ cutaneous cells down-regulated CLDN1 expression (*P* < 0.05), while CM of ANGPTL4^hi^ MBM cells did not significantly alter the expression of this TJ protein, compared to the corresponding control cells. CM of ANGPTL4^lo^ MBM cells also did not alter CLDN1 expression compared to the corresponding control cells. This suggests that ANGPTL4 contributes to melanoma cell passage through BBB junctions.

### *In vivo* functios: ANGPTL4 enhances melanoma tumorigenicity

The question if and how ANGPTL4 is involved in the shaping of the *in vivo* malignancy phenotype of melanoma cells was examined. Local tumor formation was assessed in nude mice orthotropically inoculted with CON^pQC^ or with ANGPTL4^hi^ cutaneous human melanoma cells.

**Figure 4:**
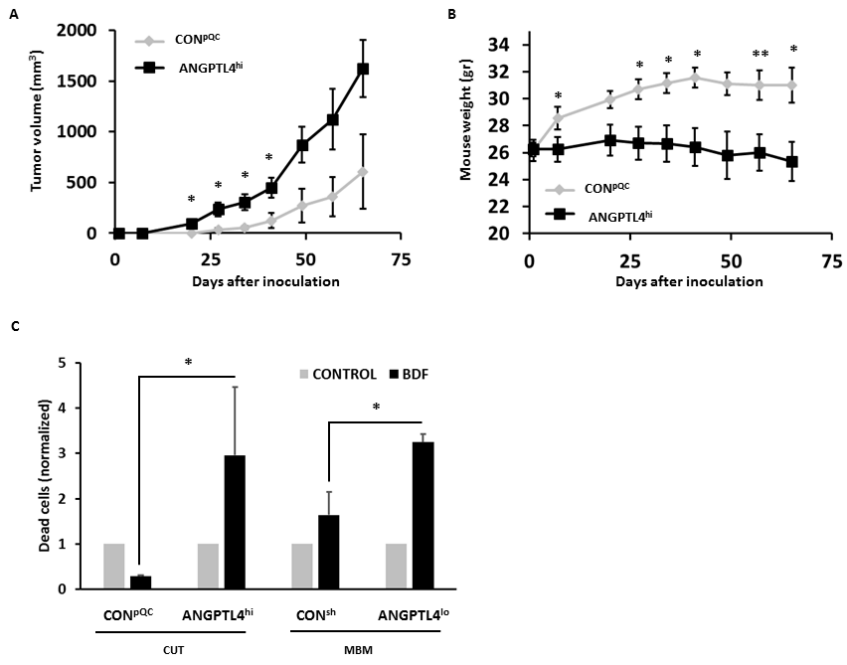
ANGPTL4 alters the tumorigenic potential of melanoma cells **A.** Volume of cutaneous tumors following subdermal implantation of CON^pQC^ vs. ANGPTL4^hi^ cutaneous cells. Tumor dimensions were measured using a caliper and volume was obtained as described in Materials and Methods. The average tumor volume + SEM is presented. *P < 0.05. **B.** Mice were weighed weekly following melanoma inoculation. The averege mouse weight + SD is presented. *P < 0.05. **C.** Melanoma cells were treated for 72 hrs with mouse BDF. Then, cells were trypsinized and cell death was determined by measuring DAPI incorporation. The bars represent the relative cell death of BDF treated cells compared to their controls + SEM obtained in one measurement in three independent experiments. *P < 0.05.

An orthotopic inoculation of ANGPTL4^hi^ cutaneous cells resulted in a significantly enhanced tumor growth compared to inoculation of CON^pQC^ cutaneous cells (*P* < 0.05) (Figure 4A). Mice inoculated with the ANGPTL4^hi^ cutaneous cells weighed less than those inoculated with the CON^pQC^ cutaneous cells, indicating the highly malignant phenotype of the desease (*P* < 0.05) (Figure 4B).

We next asked whether ANGPTL4 over-expression in cutaneous melanoma cells would influence the capacity of such cells to form spontaneous brain micro-metastasis. Nude mice were inoculated orthotopically with control (CON^pQC^) cutaneous melanoma cells or with cells overexpressing ANGPTL4 (ANGPTL4^hi^ ). The mice were killed 65 days post inoculation and anaylzed, by RT-qPCR using human specific primers, for the presence of human melanoma micro-metastastais. No significant differences were found in the incidence of MBM between the two groups: CON^pQC^ cells (brains of 10 mice out of 15 were positive for melanoma cells) and ANGPTL4^hi^ cells (brains of 7 mice out of 16 were positive for melanoma cells) .

### The effects of BDF on melanoma cell viability is ANGPTL4-dependent

Once extravasating to the target organ, metastasis propagation depends on the ability of disseminated tumor cells to survive and proliferate in their new microenvironment. Several studies showed that ANGPTL4 is a regulator of cell proliferation [28]. We previously demonstrated that BDF promote the viability of cutaneous melanoma cells but induce cell death of MBM cells [29].

In order to find out whether ANGPTL4 levels play a role in the differential susceptability of the cutaneous and MBM cells to these soluble factors, we examined the effect of BDF on the viability of melanoma cells, expressing high or low levels of ANGPTL4. ANGPTL4^hi^ and CON^pQC^ cutaneous melanoma cells and ANGPTL4^lo^ and CON^sh^ MBM cells were treated with BDF or starvation medium (control) for 72 hrs. Cell death was determined by 4’,6-Diamidino-2-phenylindole dihydrochloride (DAPI) incorporation measurement. Figure 4C demonstrates that BDF protected CON^pQC^ cutaneous cells (low ANGPTL4 expressing) from spontaneous cell death. On the other hand BDF induced cytotoxicity in ANGPTL4^hi^ cutaneous cells. These experiments lead us to conclude that a high expression of ANGPTL4 in the cutaneous cells sensitizes these melanoma cells to cytotoxicity mediated by BDF.

BDF treatment of MBM cells expressing relatively high levels of endogenous ANGPTL4 (CON^sh^) induced cell death. Treating MBM cells expressing reduced levels of ANGPTL4 (ANGPTL4^lo^) with BDF, resulted in further induction of cell death. Expression of ANGPTL4 in the MBM cells protects these melanoma cells from death exerted by BDF, increasing their sustainability in the brain microenvironment.

### Reverse Phase Protein Array (RPPA) analysis of melanoma cells differentially expressing ANGPTL4

**Figure 5:**
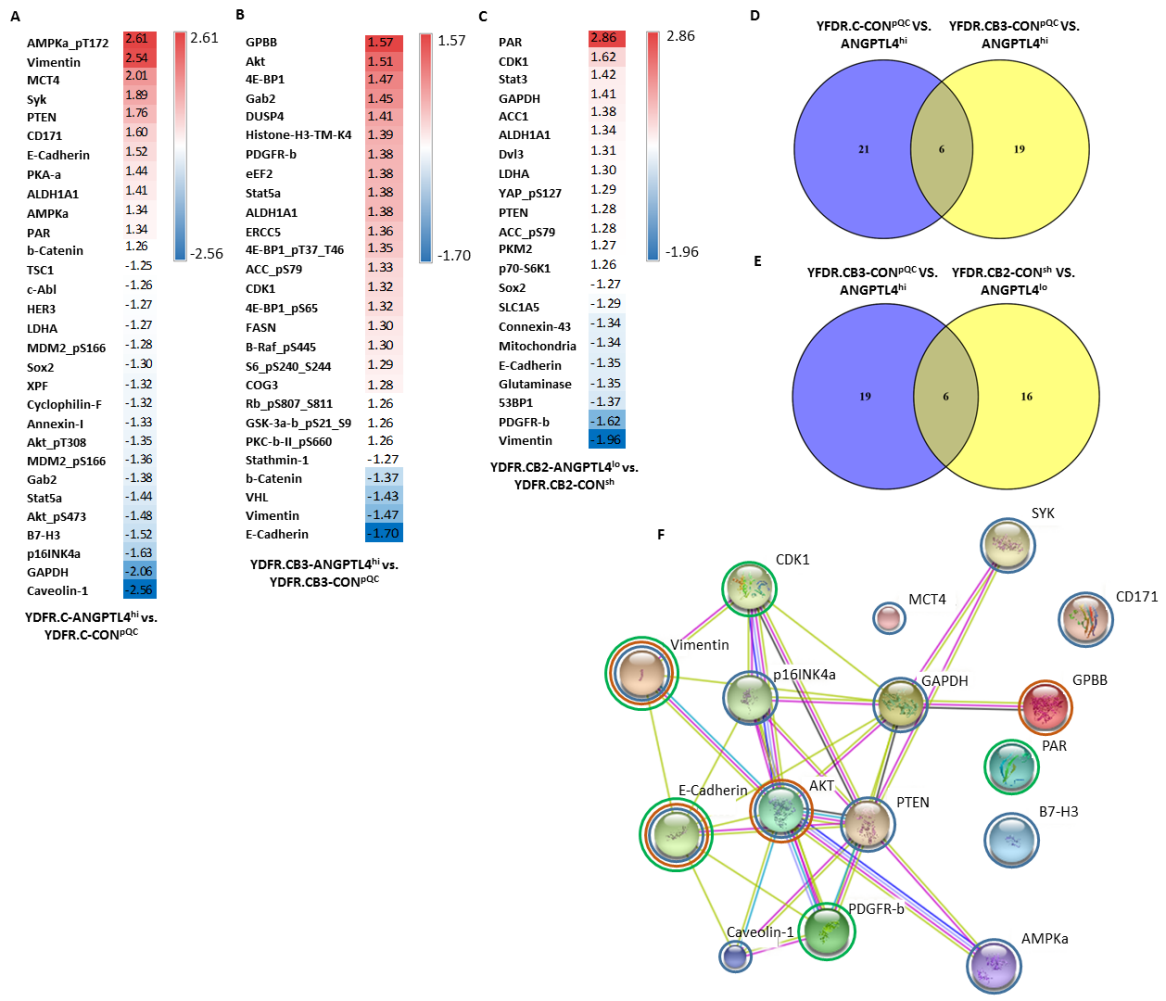
Proteomic expression analysis of ANGPTL4 high and low expressing cells The expression of 305 proteins was examined using RPPA analysis in the ANGPTL4^hi^ and ANGPTL4^lo^ cells vs. their corresponding control cells. **A.**, **B.**, **C.** Comparisons were done for each pair of cells: (A) cutaneous ANGPTL4^hi^ vs. CON^pQC^ cells, (B) brain metastasizing ANGPTL4^hi^ vs. CON^pQC^ cells and (C) brain metastasizing ANGPTL4^lo^ vs. CON^sh^ cells. The tables show the normalized expression of differentially expressed proteins with FC < -1.25 or FC>1.25. **D.**, **E.** Venn diagrams were used to compare lists of differentially expressed proteins. (D) Proteins differentially expressed in both comparisons obtained in (A) and (B). (E) Proteins differentially expressed in both comparisons obtained in (B) and (C). **F.** Protein-protein interactions of differentially expressed proteins with FC < -1.5 or FC>1.5 from all three comparisons. Circled in blue are proteins from comparison (A), circled in orange are proteins from comparison (B), and circled in green are proteins from comparison (C).

In the next set of experiments we examined the effect of ANGPTL4 over-expression or knock-down on protein expression profile in melanoma cells. To this end we compared RPPA data of ANGPTL4 overexpressing (ANGPTL4^hi^) cutaneous melanoma cells and MBM cells with the corresponding control (CON^pQC^) cells. For each pair of cells we established a list of proteins which were differentially expressed in a FC < -1.25 or FC>1.25 (Figure 5A, 5B, respectively). Identifying the differentially expressed proteins may highlight the mechanism by which ANGPTL4 regulates the malignancy phenotype of melanoma cells.

We observed 27 proteins that were differentially expressed in the cutaneous melanoma cell pair (YDFR.C-ANGPTL4^hi^ vs. YDFR.C-CON^pQC^) comparison (Figure 5A). This list of proteins includes CD171 (L1CAM), which is up-regulated (FC=1.6) in ANGPTL4^hi^ cells. L1CAM has a role in metastatic cell spreading along brain capillaries and in metastatic outgrowth [30]. Another up-regulated protein is monocarboxylate transporter 4 (MCT4; FC=2.01), a lactate transporter, whose expression was found to be increased with melanoma progression from nevi to advanced metastatic disease [31].

We observed 25 proteins that were differentially expressed in the MBM cell pair (YDFR.CB3-ANGPTL4^hi^ vs. YDFR.CB3-CON^pQC^) (Figure 5B). This list of proteins includes the von Hippel-Lindau tumor suppressor (VHL), down-regulated (FC=-1.43) in the ANGPTL4^hi^ cells.

RPPA analysis was also performed to establish the effect of ANGPTL4 knock-down on protein expression profile of the MBM cell pair (YDFR.CB2-ANGPTL4^lo^ vs. YDFR.CB2-CON^sh^). RPPA analysis yielded a list of 22 proteins that were differentially expressed in a FC < -1.25 or FC>1.25 (Figure 5C). This list of proteins include connexin-43 (FC=-1.34), whose inhibition promotes melanoma cell proliferation [32].

Venn diagram analysis was performed to provide a list of shared differentially expressed proteins that are altered in the cutaneous and MBM ANGPTL4 over-expressing cells compared to their controls (Figure 5D). 6 proteins were identified in both lists: ALDH1A1, β-catenin, E-Cadherin, Gab2, Stat5a and vimentin. These proteins are all either up-stream or down-stream to epithelial to mesenchymal transition (EMT) regulators in tumor cells [33–36]. This may imply that EMT constitutes a mechanism by which ANGPTL4 is involved in melanoma progression. Most interesting proteins are those that show an opposite trend of alteration in the cutaneous vs. the MBM pairs, in correspondence to the opposite pro/anti-malignancy effects of ANGPTL4 over-expression observed in the experiments above. Five of these proteins: E-Cadherin, GRB2-associated-binding protein 2 (Gab2), Stat5a, vimentin, and β-catenin were differentailly expressed in the two comparisons in an opposite manner.

Venn diagram analysis was also performed to provide a list of shared differentially expressed proteins that are altered following ANGPTL4 over-expression and knock-down in the metastatic variants (Figure 5E). Six proteins appeared in both comparisons: ACC_pS79, ALDH1A1, CDK1, E-Cadherin, PDGFR-beta, and Vimentin. PDGFR-beta was differentially expressed in the two comparisons in an opposite manner. These results imply again that EMT may be involved in ANGPTL4-mediated melanoma progression, due to the observed changes in the expression of these proteins, all of which are either up-stream or down-stream to EMT regulation in tumor cells [37–39].

Network of protein interactions was created for proteins differentially expressed in a FC < -1.5 or FC>1.5 from the three comparisons using STRING (Figure 5F). The biological processes ascribed to these differentially expressed proteins include: kinase activity, metabolism, proliferation and apoptosis, all of which have relevance in metastasis.

## DISCUSSION

Metastasis is the major cause leading to death from cancer. Treating metastasis is complex since frequently it has already occurred by the time the disease was diagnosed [40]. The growth and progression of metastasis is dependent on the tumor autonomous traits as well as on the interactions of the cancer cells with microenvironmental cells in their vicinity and with soluble factors released or secreted by them [40–45]. However, relatively little information exists on the interaction of constituent normal brain cells of tumor microenvironment with brain metastasis. Understanding the molecular basis for the spread of melanoma to the brain and the cellular interactions within the microenvironment is necessary for the development of novel therapeutic and prognostic solutions for this disease.

This study showed that ANGPTL4 is more highly expressed in MBM cells than in corresponding cutaneous melanoma cells in human MBM models as well as in clinical samples. Interestingly, MBM exhibited higher expression of ANGPTL4 than paired LNM. This result emphasizes the importance and selectivity of ANGPTL4 for metastasis propagation in the brain.

We have demonstrated that soluble factors in the brain microenvironment, including those secreted by microglia, astrocytes and brain endothelium, up-regulate ANGPTL4 expression. The exact soluble components of the brain which are responsible for ANGPTL4 up-regulation were not yet characterized. One of the candidate factors is TGFβ1, known to be up-regulated in the injured or inflamed brain [46–49].

The increase in ANGPTL4 expression, may, at least in part, be attributed to TGFβ1 stimulation. Cancer cells, including melanomas, tend to selectively utilize the beneficial functions of TGFβ signal transduction, including ANGPTL4 up-regulation (via the Smad signaling pathway), while eliminating the tumor suppressor function downstream to TGFβ [50, 51]. In ER negative breast tumors, TGFβ induces ANGPTL4 expression, thereby priming tumor cells for lung metastasis. This is enabled by microenvironmental TGFβ that induces or upregulates ANGPTL4 expression in disseminating tumor cells on their way to secondary metastatic sites. The upregulated ANGPTL4 enables tumor cells to disrupt vascular endothelial junctions when arriving the lung capillaries, thereby facilitating their trans-endothelial passage [50, 51]. A similar mechanism may operate in the case of MBM, as we have demonstrated that ANGPTL4 increases the ability of melanoma cells to penetrate brain endothelial cell monolayer.

To investigate the involvement of ANGPTL4 in melanoma malignancy, we have established cutaneous and MBM cells in which ANGPTL4 is overexpressed and MBM cells in whom ANGPTL4 is knocked-down.

The results demonstrated that like certain other genes [52, 53], ANGPTL4 plays a yin-yang role depending on tumor stage; it promotes malignancy of cutaneous melanoma cells and ameliorates the malignant phenotype of brain-metastasizing cells.

In the early phases of the metastatic cascade, when the tumor cells are still outside the brain microenvironment, over-expression of ANGPTL4 in the cutaneous cells promoted characteristics that would enable their invasion to the target organ, the brain microenvironment. These malignancy-promoting activities included increase in the volume of the primary tumor, increased MMP activity, promoting migration through ECM, adhesion to BEC and penetration through a brain endothelial layer towards astrocytes. Stimulation of BEC with CM of ANGPTL4 over-expressing cutaneous melanoma cells down-regulated the expression of CLDN1, a TJ protein involved in cell adhesion.

Un-expectedly and in contrast to the enhanced ability of ANGPTL4 overexpressing cutaneous cells to form local tumors, their ability to form brain metastasis was not promoted. Based on the results summarized above, the following scenario may account for these seemingly contradictory findings: In view of the fact that ANGPTL4 overexpression enhances migration and transmigration of cutaneous melanoma cells through the BBB, such cells would preferentially localize in the brain. However ANGPTL4 overexpressing cutaneous melanoma cells are sensitive to the cytotoxic activity of BDF which would diminish metastasis.

BDF, as described in the Materials and Methods section is a mixture of soluble factors released from the brain of BALB/c mice incubated for 24 or 48 hrs (depending on the experiment) at 37°C. This method of preparation was developed by Maman et al. from our laboratory [54, 55] who prepared lung derived factors which restrained the proliferation of lung metastasis. Preliminary, unpublished experiments indicated that the BDF is a protein with a molecular weight above 3.5kDa. In the future we plan to identify the active factors in the BDF, that affect melanoma cell viability and other observed phenomena (i.e. ANGPTL4 expression).

Over-expression of ANGPTL4 in the MBM cells moderates the malignancy-promoting activities. Down-regulation of ANGPTL4 in MBM cells yielded, in general, compatible results to those reported above: The malignancy phenotype of these cells was promoted. MBM cells with down-regulated levels of ANGPTL4 adhered better to BEC, secreted higher levels of active MMP-2 and penetrated more efficiently through the BBB.

The scenario taking place at later stages of metastasis when melanoma cells have already penetrated the tumor microenvironment of the brain is different. Cancer cells must be able to survive and proliferate in order to establish the metastatic foci. Cells should have the ability to avoid cell death and to promote angiogenesis, necessary for nutrient and oxygen supply. For angiogenesis to occur, endothelial cells must migrate and proliferate at the distant metastatic site [24].

MBM cells, expressing high levels of endogenous ANGPTL4 (CON^sh^ cells), are less sensitive to the viability restraining function of BDF then the same cells with down-regulated ANGPTL4 expression (ANGPTL4^lo^ cells). ANGPTL4 may thus protect such cells from BDF mediated death.

Soluble factors from MBM cells over-expressing ANGPTL4 had a growth promoting effect on BEC. Down-regulation of ANGPTL4 in the MBM cells resulted in reduced BEC viability. Therefore, ANGPTL4 expressed by MBM cells may support angiogenesis in the brain. This possibility will be further investigated in the future.

The experiments described above demonstrate that ANGPTL4 expression level has a significant involvement in melanoma-BEC interactions, and that autocrine effects of ANGPTL4 on melanoma cells may result in paracrine effects on BEC.

As indicated above, TGFβ1 induced ANGPTL4 expression in melanoma cells. Interestingly, RPPA analysis of high vs. low ANGPTL4 expressing melanoma cells demonstrated that most differentially expressed proteins are either up-stream or down-stream of the EMT cascade, which is classically controlled by the TGFβ pathway. Further investigation is aimed to elucidate the mechanism by which ANGPTL4 regulates these proteins. It is not unlikely that similarly to how ANGPTL2 and TGFβ1 positively increase the expression of each other in chronic kidney disease [56], ANGPTL4 and TGFβ1 employ such reciprocal effects on each other expression.

Taken together our study demonstrates that whereas ANGPTL4 promotes the malignancy phenotype of cutaneous melanoma cells in early stages of brain metastasis, it ameliorates the malignancy phenotype of MBM cells in the later stages of brain metastasis, when the brain tumor microenvironment plays a significant functional role in disease progression. The data presented is in agreement with studies showing that the expression and roles of ANGPTL4 are context and tumor stage dependent, and that ANGPTL4 has a diverse role in metastasis [34].

**Figure 6:**
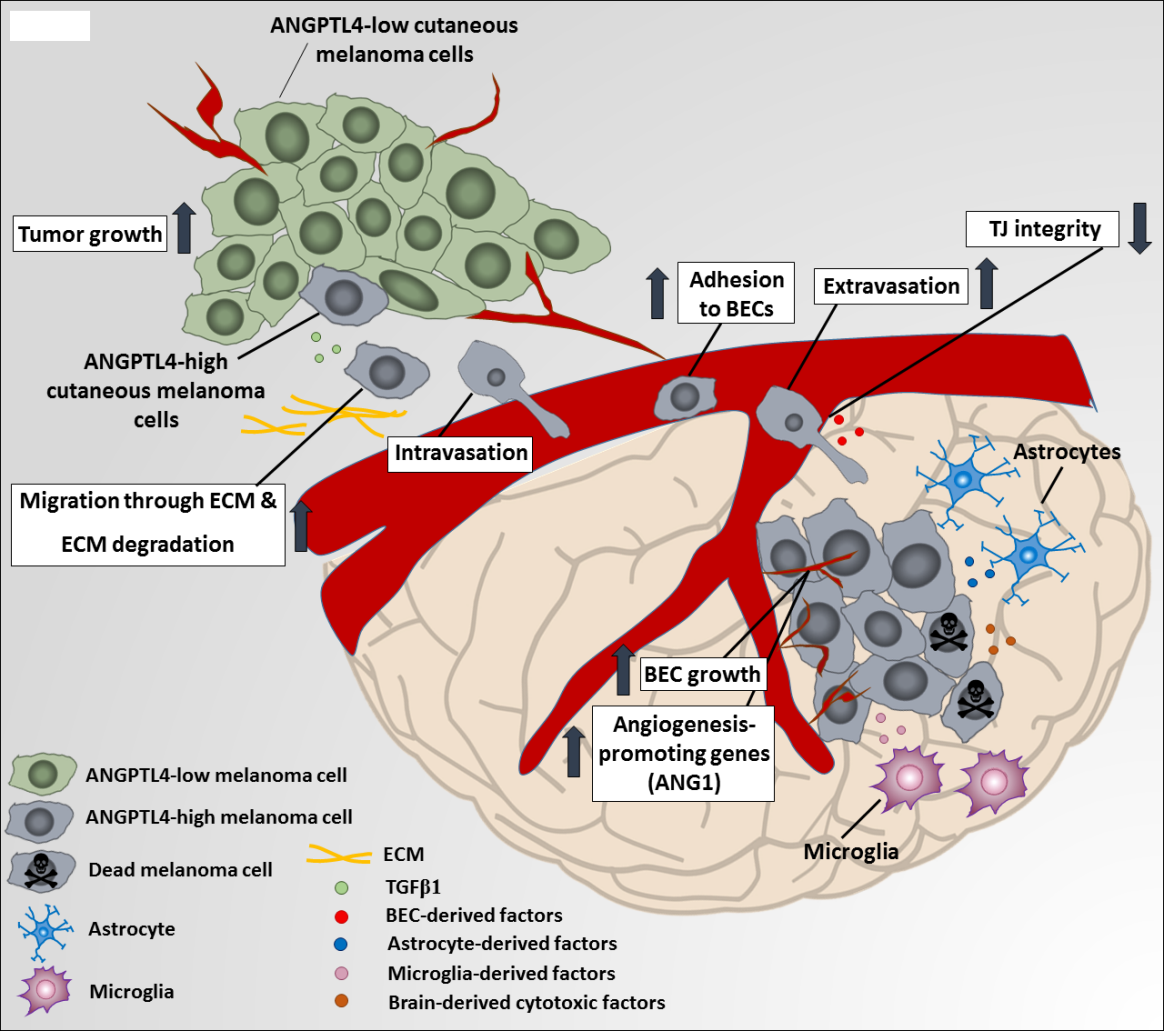
A proposed mechanism for ANGPTL4-mediated melanoma malignancy progression A soluble factor in the microenvironment of the primary tumor transforming growth factor β1 (TGFβ1) induces the expression of ANGPTL4 in primary melanoma tumor cells. ANGPTL4 enhances their ability to migrate through extracellular matrix (ECM) components and to adhere and invade brain vasculature, for example by down-regulating the expression of cell-cell adhesion tight junction (TJ) molecules such as claudin-1 (CLDN1). Once arriving the brain, brain-derived soluble factors secreted by microglia, brain endothelial cells (BEC) and astrocytes, induce ANGPTL4 expression by brain metastasizing cells, what contributes to different phenotypes, such as resistance against brain-derived cytotoxic factors, enhancement of BEC growth and induction of angiogenesis-related genes such as angiopoietin 1 (ANG1) (data not shown) in BEC subjected to factors released from brain metastasizing cells, expressing high ANGPTL4 levels.

We propose a mechanism for brain metastasis (illustrated in Figure 6) whereby a soluble factor in the microenvironment of the primary tumor (e.g. TGFβ1) induces the expression of intracellular or extracellular proteins (e.g. ANGPTL4) in cells of the primary tumor. These cells are able to migrate through ECM, adhere to and invade brain vasculature, for example by down-regulating the expression of cell-cell adhesion TJ molecules such as CLDN1 [57, 58]. Once in the brain, angiogenesis promotion, endothelial proliferation and survival may have a higher priority rather than characteristics such as adhesion and invasion. Therefore in MBM cells, high expression of ANGPTL4 (induced by brain-derived soluble factors) may contribute to different phenotypes, such as resistance against brain-derived cytotoxic factors, enhancement of BEC growth and induction of angiogenesis-related genes such as ANG1 (data not shown) in BEC subjected to factors released from MBM cells which express high levels of ANGPTL4.

## MATERIALS AND METHODS

### Animals

Male BALB/c mice were purchased from Tel Aviv University animal facility. Male athymic nude mice (BALB/c background) were purchased from Harlan Laboratories Limited (Israel). The mice were housed and maintained in laminar flow cabinets under specific pathogen-free conditions in the animal quarters of Tel-Aviv University and in accordance with current regulations and standards of the Tel-Aviv University Institutional Animal Care and Use Committee. The mice were used when they were 7-8 weeks old.

### Cell culture

The production and maintenance of cutaneous human melanoma variant (YDFR.C) and MBM variants (YDFR.CB2 and YDFR.CB3) were described previously [8].

Novel xenograft models were obtained from the parental cell lines UCLA-SO-M12, UCLA-SO-M16 and DP-0574-Me (JWCI) as we described previously for the YDFR cells [8], and consist of cutaneous variants (C variants) and brain macro-metastatic variants (CB1 and CB2 variants; the number represents the number of cycles of re-inoculation into the left ventricle of the heart as described previously [8]). Human embryonic kidney 293T cells were maintained as described previously [10]. Immortalized human brain microvascular endothelial cells (hCMEC/D3) were kindly provided by Dr. Clara Nahmias and Prof. Pierre-Olivier Couraud (Inserm, U1016, Institut Cochin, Paris, France) and were maintained as described by Weksler et al [59]. Human astrocytes were maintained as described previously [5]. Immortalized human microglia-SV40 cell line was purchased from ABM (ABM, Milton, Canada) and were maintained on 100μg/ml collagen I, rat tail (BD Biosciences, Bedford, MA, USA) in 10% fetal calf serum (FCS) supplemented Prigrow III medium (ABM).

0.5% FCS supplemented medium was used for starvation in all the experiments, unless indicated otherwise. Cells were routinely cultured in humidified air with 5% CO2 at 37°C.

### Western blotting

Cells were plated in growth medium for 24 hrs. For the detection of secreted ANGPTL4, supernatants were collected, centrifuged at 1400 rpm for 5 min and filtered (0.45µm). For the detection of intracellular ANGPTL4, the plated cells were washed twice with ice-cold PBSX1 and lysed (20mM Tris pH 8, 250mM NaCl, 0.5% Nonident p-40 (NP-40), 5mM EDTA pH 8, 4mM EGTA pH 8, 20mM sodium phosphate pH 7.6, 3mM β glycerol phosphate, 2mM sodium orthovanadate (Na3VO4), 5mM NaF, 10mM sodium pyrophosphate pH 7.6, 2 µg/ml aprotinin, 2 µg/ml leupeptin and 1mM phenylmethylsulfonyl (PMSF) supplemented with DDW). Cell lysates were incubated for 20 min on ice and cleared by centrifuging at 16,000g for 20 min at 4°C. Pierce BCA protein assay kit (Thermo Scientific, Rockford, IL, USA) was used to determine protein concentration. After the addition of Laemmli sample buffer, the lysates or supernatants were resolved on SDS-PAGE, and transferred onto nitrocellulose membrane. The total amount of protein in the lanes was assessed by Ponceau staining prior to blocking of the membrane. The membranes were blocked at room temperature with 3% BSA diluted in TBS-Tween for 1 hr. For detection of the target proteins, membranes were incubated with relevant primary antibodies: Anti-human ANGPTL4 antibody (Ab) (1:350, R&D Systems, Inc., Minneapolis, MA, USA) and rabbit polyclonal to beta Tubulin (1:500, Abcam, Cambridge, UK). Horseradish peroxidase-conjugated donkey anti-goat or goat anti-rabbit (1:10000, Jackson ImmunoResearch Laboratories, West Grove, PA, USA) were used as secondary Ab.

The bands were visualized by chemoluminescence-ECL reactions and autoradiography by exposure to Fuji film. The amount of the relevant protein in the lanes was estimated by densitometry using Quantity One Image software.

### Flow cytometry

CLDN1 expression was analyzed by monoclonal anti-human CLDN1 Ab (1μg/sample, R&D Systems), using flow cytometry as described previously [9]. Fluorescein isothiocyanate (FITC)-conjugated goat anti-rat IgG (1:50, Jackson ImmunoResearch Laboratories) was used as secondary Ab.

Antigen expression was determined using Becton Dickinson FACSort (Becton Dickinson Mountain View, CA, USA) and CellQuest software. Dead cells were gated out from the analysis.

### Immunohistochemistry (IHC)

Cutaneous melanoma patients were included in the study at John Wayne Cancer Institute under the MORD-RTPCR-0995 protocol approved by the Western Institutional Review Board (WIRB Protocol #20072107). Informed consent was obtained from all subjects and the experiments were performed according to the principles set out in the WMA Declaration of Helsinki and the NIH Belmont Report. Tissue specimens were de-identified and coded according to HIPAA recommendations to ensure the confidentiality of the patients.

IHC was performed on 5µm human melanoma sections. Slides were deparaffinized, rehydrated, and then antigen retrieval was performed using 10mM sodium citrate buffer (pH 6.0) in a boiling water bath for 20 min. The sections were then incubated in 10g/L trichloroisocyanuric acid (Sigma-Aldrich) solution for 30 min at RT to bleach melanin [60]. After rinse with tap water, endogenous peroxidase was blocked with hydrogen peroxide for 10 min and nonspecific binding was blocked with Protein Block serum-free solution (Dako, Glostrup, Denmark) for 10 min. The sections were incubated overnight with the anti-ANGPTL4 rabbit polyclonal Ab (Proteintech Group, Inc, Rosemont, IL, USA) at a dilution of 1:100 in a 4°C humidified chamber. Visualization was performed using LSAB2 system-HRP (Dako) and Liquid DAB+ Substrate Chromogen System (Dako) according to the manufacturer's instructions. After visualization, the sections were counterstained with Gill's hematoxylin (Sigma-Aldrich) for 1.5 min at RT, dehydrated, and mounted. After the IHC, photographs were taken using a Nikon Eclipse Ti microscope and NIS elements software (Nikon, Melville, NY, USA) and analyzed by ImageJ software (National Institute of Health, version 1.50i). The expression of ANGPTL4 was quantified using H score system [61], which considers both the intensity and percentage of positive cells. The score was calculated using the formula 1x(% of 1+ cells)+2x(% of 2+ cells)+3x(% of 3+ cells).

### Reverse phase protein array (RPPA)

1×10^6^ melanoma cells were seeded for 24 hrs. Then cells were harvested by Trypsin EDTA (Biological Industries). Cell pellet was washed twice with phosphate-buffered saline (PBS) and lysed (1% Triton X-100, 50mM HEPES, pH 7.4, 150mM NaCl, 1.5mM MgCl2, 1mM EGTA, 100mM NaF, 10mM Na pyrophosphate, 1mM Na3VO4, 10% glycerol, protease and phosphatase inhibitors). Protein concentration was adjusted to 1-1.5µg/µl and denatured by 1% SDS. Cell lysates were two-fold-serial diluted for 5 dilutions (from undiluted to 1:16 dilution) and arrayed on nitrocellulose-coated slides in 11×11 format. Samples were probed with Ab by CSA amplification approach and visualized by DAB colorimetric reaction.

Slides were scanned on a flatbed scanner to produce 16-bit tiff images. Spots from tiff images were identified and the density was quantified by Array-Pro Analyzer. Relative protein levels for each sample were determined by interpolation of each dilution curve from the “standard curve” (supercurve) of the slide (Ab). Supercurve is constructed by a script in R written by Bioinformatics (“Supercurve Fitting” developed by the Department of Bioinformatics and Computational Biology in MD Anderson Cancer Center, http://bioinformatics.mdanderson.org/Software/supercurve). Each dilution curve was fitted with a logistic model. This fits a single curve using all the samples (i.e., dilution series) on a slide with the signal intensity as the response variable and the dilution steps are independent variable. The fitted curve (“supercurve”) was plotted with the signal intensities on the Y-axis and the relative log2 concentration of each protein on the X-axis using the non-parametric, monotone increasing B-spline model. During the process, the raw spot intensity data were adjusted to correct spatial bias before model fitting. A QC metric was returned for each slide to help determine the quality of the slide: if the score is less than 0.8 on a 0-1 scale, the slide was dropped. In most cases, the staining was repeated to obtain a high quality score. The protein concentrations of each set of slides were then normalized by median polish, which was corrected across samples by the linear expression values using the median expression levels of all Ab experiments to calculate a loading correction factor for each sample.

These values (given as log2 values) are defined as Supercurve Log2 (Raw) values. All the data points were normalized for protein loading and transformed to linear values.

The identification of proteins differentially expressed in the different comparisons of overexpression/down-regulation compared to control (fold change (FC < -1.25 or FC >1.25) was performed.

Venny tool was used to compare between differentially expressed genelists (http://bioinfogp.cnb.csic.es/tools/venny/).

Network of protein interactions between differentially expressed proteins (FC < -1.5 or FC>1.5) was generated using STRING (http://string-db.org).

### Reverse transcription quantitative real-time PCR (RT-qPCR)

cDNA extraction and RT-qPCR were performed as described previously [8]. For mRNA amplification, primers were designed based on the GenBank Nucleotide Database of the NCBI website: ANGPTL4: S-5‘-CTGCGAATTCAGCATCTGCAA-3‘, AS-5‘-CCTCAGGTCTAGGTGCTTGT-3‘; CLDN1: S-5‘-AAGATGAGGATGGCTGTCATT-3‘, AS-5‘ATACCATGCTGTGGCAACTAA-3‘; human RS9: S-5‘-CGGAGACCCTTCGAGAAATCT-3‘, AS-5‘-GCCCATACTCGCCGATCA-3‘. Amplification reactions were performed with SYBR Green I (Thermo fisher scientific, ABgene, Germany) in duplicates in Rotor-gene 6000TM (Corbett life science, Australia) and Rotor-Gene Q software. PCR amplification was performed over 40 cycles (95°C for 15 sec, 59°C for 20 sec, 72°C for 15 sec).

### Construction of the expression vector and stable over-expression of ANGPTL4

The over-expression construct of human *ANGPTL4* (NM_139314) was created by PCR amplification of genomic DNA by Phusion^®^ High-Fidelity DNA polymerase (Thermo Fisher Scientific), using the following primers (designed based on the GenBank Nucleotide Database of the NCBI website): ANGPTL4: S-5’-TCTCTCACCGGGTATGAGCGGTGCTCCGACGGCC-3’, AS-5’-GTGTCTTAATTAACTAGGAGGCTGCCTCTGCTGC-3’ The generated fragment was digested with AgeI and PacI and ligated into the corresponding sites of pQCXIP vector (Clontech Laboratories, Inc., Mountain View, CA, USA). PCR products of *ANGPTL4* were sequenced and found to be identical to the published sequence. Production of infectious viruses and melanoma infection were performed as described previously [10].

To produce mCherry expressing cells, melanoma cells were similarly transduced with a pQCXIN-*mCherry* plasmid and were selected using 800μg/ml G418 Sulfate (A.G. Scientific, Inc., San Diego, CA, USA).

### ANGPTL4 down-regulation in melanoma cells

The down-regulation of ANGPTL4 was established using a mixture of four different pGIPZ vectors containing shRNA sequences targeting ANGPTL4 mRNA (NM_139314) (RHS4430-200184490, RHS4430-200245716, RHS4430-200246249 and RHS4430-200243106; Thermo Fisher Scientific). A sh-non-silencing pGIPZ vector (RHS4531) was used as a negative control.

To produce the infectious viruses, the 293T packaging cell line was co-transfected using the calcium phosphate method with the lentiviral plasmids shANGPTL4-pGIPZ or the sh-non-silencing-pGIPZ, packaging plasmid pCMV∆R8.2 and envelope plasmid pVSV-G. After 48 hrs, the virus particles in the medium were collected and filtered (0.45μm; Whatman GmbH).

Melanoma cells, seeded 24 hrs before infection, were infected in the presence of 8µg/ml polybrene and the virus-containing medium, which was afterwards replaced with fresh growth medium. After 72 hrs 2µg/ml puromycin (InvivoGen, San Diego, CA, USA) was added for additional 7 days to select stably infected cell populations. After selection, puromycin was continuously added to the culture.

### Preparation of melanoma or brain cell CM

Melanoma, microglia, astrocyte or BEC were cultured for 24 hrs. The cells were washed and starved for 24 hrs. CM was collected, centrifuged for 5 min at 1400 rpm and filtered (0.45μm).

### Stimulation of melanoma cells with BDF for cell viability analysis

Brains of BALB/c mice were harvested, minced to pieces in starvation medium and put on non-tissue culture dishes in 10ml of the same media for 48 hrs at 37°C, to allow the secretion of BDF. Then, supernatantes were collected, centrifuged for 5 min at 1400 rpm, filtered (0.45μm) and further diluted to a concentration of 1.6mg/ml. Melanoma cells, cultured 24 hrs prior to the experiment, were treated with BDF for 72 hrs at 37°C. Starvation medium was added to control melanoma cells. After 72 hrs, cell death was determined following the addition of 5µl of 5mg/ml DAPI using S1000Exi flow cytometer (stratedigm, San Jose, CA, USA) and FlowJo software.

### Stimulation of melanoma cells with tumor growth factor β1 (TGFβ1)

Melanoma cells were starved for 1 hr, then stimulated for 4 hrs with 5ng/ml [62] recombinant human TGFβ1 (PeproTech Inc., Rocky Hill, NJ, USA) at 37°C. Control cells were treated with the same medium without TGFβ1.

### Stimulation of melanoma cells with BDF for ANGPTL4 expression analysis

BDF were prepared as mentioned above in 0.5% bovine serum albumin (BSA) supplemented RPMI-1640, after 24 hrs of incubation at 37°C. Melanoma cells, cultured 24 hrs prior to the experiment, were treated with BDF for 24 hrs at 37°C. 0.5% BSA supplemented RPMI-1640 was added to control cells. Cells were analyzed for ANGPTL4 expression using RT-qPCR.

### Viability assay (XTT)

Cell Proliferation Kit (XTT, Biological industries) was used according to the manufactures’ instructions. To obtain the relative cell viability, the optical density (OD) of the treated cells (in each time point) was divided by the OD of the non-treated cells at the same time point of the experiment.

### Adhesion to BEC

Adhesion of melanoma cells to BEC was performed as described previously [10]. Adhesion of GFP-expressing cells was measured at wavelength of 490/530. Adhesion of mCherry-expressing cells was measured at wavelength of 590/645. To obtain the cell relative adhesion capacity, the OD of the adherent cells was divided by the OD of the total cells plated.

### Migration through extracellular matrix

1×10^5^ melanoma cells were loaded onto collagen-coated transwell inserts (8μm; Corning Costar Corp., New York, NY, USA) and allowed to migrate for 24 hrs towards starvation medium. Then, the upper side of the apical chamber was scraped gently with cotton swab, to remove non-migrating cells. Cells at the bottom side of the transwell inserts were fixed with ice-cold methanol for 5 minutes. Migrating cells were stained with Dif-stain Kit (Kaltek, Padova, Italy), according to the manufacturer‘s instructions. The number of melanoma cells transmigrating to the underside of the membrane was determined in each experiment by counting at least five independent fields in duplicates using Olympus^IX53^ inverted microscope (Olympus, Center Valley, PA, USA).

### Transendothelial migration through a BBB model

*In vitro* BBB models consisting of BEC co-cultured with astrocytes are utilized for studying the properties of the BBB [63].

5×10^4^ BEC were loaded onto collagen-coated transwell inserts (8μm; Corning Costar Corp.) and allowed to create a monolayer for 48 hrs (illustrated in Figure 2G). Simultaneously, 5×10^4^ astrocytes were separately plated at the bottom of Poly-L-Lysine-coated 24-wells. 24 hrs later astrocytes were washed and starved for another 24 hrs, to allow secretion of astrocyte-derived soluble factors into the medium. 48 hrs after seeding of BEC and astrocytes, 1×10^5^ mCherry-expressing melanoma cells were loaded onto the endothelial monolayer and allowed to migrate towards the astrocytes for 24 hrs. Then, the upper side of the apical chamber was scraped gently with cotton swab, to remove non-migrating cells. Cells at the bottom side of the transwell inserts were fixed with 4% paraformaldehyde and mounted with DAPI Fluoromount-G (SouthernBiotech, Birmingham, AL, USA). The number of melanoma cells transmigrating to the underside of the membrane was determined in each experiment by counting at least five independent fields under fluorescence microscopy in duplicates using Olympus^IX53^ inverted microscope (Olympus).

### Gelatin zymography analysis

4.5×10^5^ melanoma cells were plated in 24-well plates for 24 hrs, then growth medium was removed and replaced by 500µl serum-free RPMI-1640 medium for additional 24 hrs. To determine MMP-2 activity, CM was collected and subjected to gelatin and collagen zymography assay as described previously [64].

### Orthotopic inoculation of tumor cells and *in vivo* tumorigenicity and metastasis formation assays

To generate subcutaneous tumors, 1×10^6^ cells in 100μl of 5% FCS supplemented RPMI-1640 medium were inoculated subdermally into the right thigh of male nude-BALB/c mice.

To test the tumorigenic properties of derived cell lines, subcutaneous tumors were measured once a week using a caliper. Tumor volume was obtained by the ellipsoid volume calculation formula 0.5X(lengthXwidth^2^) [65, 66]. Mice were weighed weekly. Mice were killed when moribund, and brains were harvested and immediately frozen and stored at -70°C, until used for RNA extraction. Detection of human cells (micro-metastases) in mouse brain by RT-qPCR was performed as described previously [8].

### Statistical analysis

Paired or unpaired Student t test was used to compare *in vitro* and *in vivo* results.

For the IHC experiments, statistical differences on the H-score values were analysed by using the Kruskal-Wallis test among the groups. Additionally, the Steel-Dwass method was used to further compare between groups that were considered statistically significant. The statistical analyses were performed with EZR (Saitama Medical Centre, Jichi Medical University, Saitama, Japan, version 1.32), a graphical user interface for R (The R Foundation for Statistical Computing, Vienna, Austria).

## Abbreviations

ANGPTL4: angiopoietin-like 4
BBB: blood-brain barrier
BDF: brain-derived factors
BEC: brain endothelial cells
BSA: bovine serum albumin
CLDN1: claudin-1
CM: conditioned medium
DAPI: 4′,6-diamidino-2-phenylindole dihydrochloride
ECM: extracellular matrix
FCS: fetal calf serum
FITC: fluorescein isothiocyanate
IHC: Immunohistochemistry
LNM: lymph node metastasis
MBM: melanoma brain metastasis
MMP-2: matrix metalloproteinase-2
OD: optical density
PBS: phosphate-buffered saline
PRM: primary melanoma
RPPA: reverse phase protein array
RS9: ribosomal protein S9
RT-qPCR: quantitative real-time PCR
SD: standard deviation
SEM: standard error of the mean
TEM: transendothelial migration
TGFβ1: transforming growth factor beta 1
TJ: tight junction

## References

[1] Marzese DM, Witz IP, Kelly DF, Hoon DS (2015). Epigenomic landscape of melanoma progression to brain metastasis: unexplored therapeutic alternatives. Epigenomics.

[2] Gupta G, Robertson AG, MacKie RM (1997). Cerebral metastases of cutaneous melanoma. Br J Cancer.

[3] Bafaloukos D, Gogas H (2004). The treatment of brain metastases in melanoma patients. Cancer Treat Rev.

[4] Yano S, Shinohara H, Herbst RS, Kuniyasu H, Bucana CD, Ellis LM, Davis DW, McConkey DJ, Fidler IJ (2000). Expression of vascular endothelial growth factor is necessary but not sufficient for production and growth of brain metastasis. Cancer Res.

[5] Klein A, Schwartz H, Sagi-Assif O, Meshel T, Izraely S, Ben Menachem S, Bengaiev R, Ben-Shmuel A, Nahmias C, Couraud PO, Witz IP, Erez N (2015). Astrocytes facilitate melanoma brain metastasis via secretion of IL-23. J Pathol.

[6] Fitzgerald DP, Palmieri D, Hua E, Hargrave E, Herring JM, Qian Y, Vega-Valle E, Weil RJ, Stark AM, Vortmeyer AO, Steeg PS (2008). Reactive glia are recruited by highly proliferative brain metastases of breast cancer and promote tumor cell colonization. Clin Exp Metastasis.

[7] Yao Y, Ye H, Qi Z, Mo L, Yue Q, Baral A, Hoon DS, Vera JC, Heiss JD, Chen CC, Hua W, Zhang J, Jin K (2016). B7-H4(B7x)-Mediated Cross-talk between Glioma-Initiating Cells and Macrophages via the IL6/JAK/STAT3 Pathway Lead to Poor Prognosis in Glioma Patients. Clin Cancer Res.

[8] Izraely S, Sagi-Assif O, Klein A, Meshel T, Tsarfaty G, Pasmanik-Chor M, Nahmias C, Couraud PO, Ateh E, Bryant JL, Hoon DS, Witz IP (2012). The metastatic microenvironment: brain-residing melanoma metastasis and dormant micrometastasis. Int J Cancer.

[9] Izraely S, Klein A, Sagi-Assif O, Meshel T, Tsarfaty G, Hoon DS, Witz IP (2010). Chemokine-chemokine receptor axes in melanoma brain metastasis. Immunol Lett.

[10] Izraely S, Sagi-Assif O, Klein A, Meshel T, Ben-Menachem S, Zaritsky A, Ehrlich M, Prieto VG, Bar-Eli M, Pirker C, Berger W, Nahmias C, Couraud PO (2015). The metastatic microenvironment: Claudin-1 suppresses the malignant phenotype of melanoma brain metastasis. Int J Cancer.

[11] Klein A, Sagi-Assif O, Meshel T, Telerman A, Izraely S, Ben-Menachem S, Bayry J, Marzese DM, Ohe S, Hoon DSB, Erez N, Witz IP (2017). CCR4 is a determinant of melanoma brain metastasis. Oncotarget.

[12] Padua D, Zhang XH, Wang Q, Nadal C, Gerald WL, Gomis RR, Massague J (2008). TGFbeta primes breast tumors for lung metastasis seeding through angiopoietin-like 4. Cell.

[13] Goh YY, Pal M, Chong HC, Zhu P, Tan MJ, Punugu L, Tan CK, Huang RL, Sze SK, Tang MB, Ding JL, Kersten S, Tan NS (2010). Angiopoietin-like 4 interacts with matrix proteins to modulate wound healing. J Biol Chem.

[14] Tan MJ, Teo Z, Sng MK, Zhu P, Tan NS (2012). Emerging roles of angiopoietin-like 4 in human cancer. Mol Cancer Res.

[15] Nakayama T, Hirakawa H, Shibata K, Abe K, Nagayasu T, Taguchi T (2010). Expression of angiopoietin-like 4 in human gastric cancer: ANGPTL4 promotes venous invasion. Oncol Rep.

[16] Bos PD, Zhang XH, Nadal C, Shu W, Gomis RR, Nguyen DX, Minn AJ, van de Vijver MJ, Gerald WL, Foekens JA, Massague J (2009). Genes that mediate breast cancer metastasis to the brain. Nature.

[17] Galaup A, Cazes A, Le Jan S, Philippe J, Connault E, Le Coz E, Mekid H, Mir LM, Opolon P, Corvol P, Monnot C, Germain S (2006). Angiopoietin-like 4 prevents metastasis through inhibition of vascular permeability and tumor cell motility and invasiveness. Proc Natl Acad Sci U S A.

[18] Li Y, Foster W, Deasy BM, Chan Y, Prisk V, Tang Y, Cummins J, Huard J (2004). Transforming growth factor-beta1 induces the differentiation of myogenic cells into fibrotic cells in injured skeletal muscle: a key event in muscle fibrogenesis. Am J Pathol.

[19] Perrot CY, Javelaud D, Mauviel A (2013). Insights into the Transforming Growth Factor-beta Signaling Pathway in Cutaneous Melanoma. Annals of dermatology.

[20] Yamaguchi H, Wyckoff J, Condeelis J (2005). Cell migration in tumors. Curr Opin Cell Biol.

[21] Cazes A, Galaup A, Chomel C, Bignon M, Brechot N, Le Jan S, Weber H, Corvol P, Muller L, Germain S, Monnot C (2006). Extracellular matrix-bound angiopoietin-like 4 inhibits endothelial cell adhesion, migration, and sprouting and alters actin cytoskeleton. Circ Res.

[22] Denkins Y, Reiland J, Roy M, Sinnappah-Kang ND, Galjour J, Murry BP, Blust J, Aucoin R, Marchetti D (2004). Brain metastases in melanoma: roles of neurotrophins. Neuro Oncol.

[23] Bouleti C, Mathivet T, Coqueran B, Serfaty JM, Lesage M, Berland E, Ardidie-Robouant C, Kauffenstein G, Henrion D, Lapergue B, Mazighi M, Duyckaerts C, Thurston G (2013). Protective effects of angiopoietin-like 4 on cerebrovascular and functional damages in ischaemic stroke. European heart journal.

[24] Lamalice L, Le Boeuf F, Huot J (2007). Endothelial cell migration during angiogenesis. Circ Res.

[25] Deryugina EI, Quigley JP (2006). Matrix metalloproteinases and tumor metastasis. Cancer Metastasis Rev.

[26] Bjorklund M, Koivunen E (2005). Gelatinase-mediated migration and invasion of cancer cells. Biochim Biophys Acta.

[27] Guo L, Li SY, Ji FY, Zhao YF, Zhong Y, Lv XJ, Wu XL, Qian GS (2014). Role of Angptl4 in vascular permeability and inflammation. Inflamm Res.

[28] Huang Z, Xie J, Lin S, Li S, Huang Z, Wang Y, Ye J (2016). The downregulation of ANGPTL4 inhibits the migration and proliferation of tongue squamous cell carcinoma. Arch Oral Biol.

[29] Klein A, Sagi-Assif O, Izraely S, Meshel T, Pasmanik-Chor M, Nahmias C, Couraud PO, Erez N, Hoon DS, Witz IP (2012). The metastatic microenvironment: Brain-derived soluble factors alter the malignant phenotype of cutaneous and brain-metastasizing melanoma cells. Int J Cancer.

[30] Valiente M, Obenauf AC, Jin X, Chen Q, Zhang XH, Lee DJ, Chaft JE, Kris MG, Huse JT, Brogi E, Massague J (2014). Serpins promote cancer cell survival and vascular co-option in brain metastasis. Cell.

[31] Ho J, de Moura MB, Lin Y, Vincent G, Thorne S, Duncan LM, Hui-Min L, Kirkwood JM, Becker D, Van Houten B, Moschos SJ (2012). Importance of glycolysis and oxidative phosphorylation in advanced melanoma. Mol Cancer.

[32] Tittarelli A, Guerrero I, Tempio F, Gleisner MA, Avalos I, Sabanegh S, Ortiz C, Michea L, Lopez MN, Mendoza-Naranjo A, Salazar-Onfray F (2015). Overexpression of connexin 43 reduces melanoma proliferative and metastatic capacity. Br J Cancer.

[33] Chen C, Wei Y, Hummel M, Hoffmann TK, Gross M, Kaufmann AM, Albers AE (2011). Evidence for epithelial-mesenchymal transition in cancer stem cells of head and neck squamous cell carcinoma. PLoS One.

[34] Chong HC, Tan CK, Huang RL, Tan NS (2012). Matricellular proteins: a sticky affair with cancers. J Oncol.

[35] Talati PG, Gu L, Ellsworth EM, Girondo MA, Trerotola M, Hoang DT, Leiby B, Dagvadorj A, McCue PA, Lallas CD, Trabulsi EJ, Gomella L, Aplin AE (2015). Jak2-Stat5a/b Signaling Induces Epithelial-to-Mesenchymal Transition and Stem-Like Cell Properties in Prostate Cancer. Am J Pathol.

[36] Wang Y, Sheng Q, Spillman MA, Behbakht K, Gu H (2012). Gab2 regulates the migratory behaviors and E-cadherin expression via activation of the PI3K pathway in ovarian cancer cells. Oncogene.

[37] Chou CC, Lee KH, Lai IL, Wang D, Mo X, Kulp SK, Shapiro CL, Chen CS (2014). AMPK reverses the mesenchymal phenotype of cancer cells by targeting the Akt-MDM2-Foxo3a signaling axis. Cancer Res.

[38] Gotzmann J, Fischer AN, Zojer M, Mikula M, Proell V, Huber H, Jechlinger M, Waerner T, Weith A, Beug H, Mikulits W (2006). A crucial function of PDGF in TGF-beta-mediated cancer progression of hepatocytes. Oncogene.

[39] de Souza Palma C, Grassi ML, Thome CH, Ferreira GA, Albuquerque D, Pinto MT, Ferreira Melo FU, Kashima S, Covas DT, Pitteri SJ, Faca VM (2016). Proteomic Analysis of Epithelial to Mesenchymal Transition (EMT) Reveals Cross-talk between SNAIL and HDAC1 Proteins in Breast Cancer Cells. Mol Cell Proteomics.

[40] Fidler IJ, Kripke ML (2015). The challenge of targeting metastasis. Cancer Metastasis Rev.

[41] Witz IP, Levy-Nissenbaum O (2006). The tumor microenvironment in the post-PAGET era. Cancer Lett.

[42] Kopfstein L, Christofori G (2006). Metastasis: cell-autonomous mechanisms versus contributions by the tumor microenvironment. Cell Mol Life Sci.

[43] Weinberg RA (2008). Coevolution in the tumor microenvironment. Nat Genet.

[44] Gupta GP, Massague J (2006). Cancer metastasis: building a framework. Cell.

[45] Witz IP (2008). Tumor-microenvironment interactions: dangerous liaisons. Adv Cancer Res.

[46] Dobolyi A, Vincze C, Pal G, Lovas G (2012). The neuroprotective functions of transforming growth factor beta proteins. Int J Mol Sci.

[47] Doyle KP, Cekanaviciute E, Mamer LE, Buckwalter MS (2010). TGFbeta signaling in the brain increases with aging and signals to astrocytes and innate immune cells in the weeks after stroke. J Neuroinflammation.

[48] Lindholm D, Castren E, Kiefer R, Zafra F, Thoenen H (1992). Transforming growth factor-beta 1 in the rat brain: increase after injury and inhibition of astrocyte proliferation. J Cell Biol.

[49] Krupinski J, Kumar P, Kumar S, Kaluza J (1996). Increased expression of TGF-beta 1 in brain tissue after ischemic stroke in humans. Stroke.

[50] Padua D, Massague J (2009). Roles of TGFbeta in metastasis. Cell Res.

[51] Drabsch Y, ten Dijke P (2011). TGF-beta signaling in breast cancer cell invasion and bone metastasis. J Mammary Gland Biol Neoplasia.

[52] Roy N, Malik S, Villanueva KE, Urano A, Lu X, Von Figura G, Seeley ES, Dawson DW, Collisson EA, Hebrok M (2015). Brg1 promotes both tumor-suppressive and oncogenic activities at distinct stages of pancreatic cancer formation. Genes Dev.

[53] Lebrun JJ (2012). The Dual Role of TGFbeta in Human Cancer: From Tumor Suppression to Cancer Metastasis. ISRN Mol Biol.

[54] Maman S, Edry-Botzer L, Sagi-Assif O, Meshel T, Yuan W, Lu W, Witz IP (2013). The metastatic microenvironment: lung-derived factors control the viability of neuroblastoma lung metastasis. Int J Cancer.

[55] Maman S, Sagi-Assif O, Yuan W, Ginat R, Meshel T, Zubrilov I, Keisari Y, Lu W, Lu W, Witz IP (2017). The Beta Subunit of Hemoglobin (HBB2/HBB) Suppresses Neuroblastoma Growth and Metastasis. Cancer Res.

[56] Morinaga J, Kadomatsu T, Miyata K, Endo M, Terada K, Tian Z, Sugizaki T, Tanigawa H, Zhao J, Zhu S, Sato M, Araki K, Iyama K (2016). Angiopoietin-like protein 2 increases renal fibrosis by accelerating transforming growth factor-beta signaling in chronic kidney disease. Kidney Int.

[57] Cichon C, Sabharwal H, Ruter C, Schmidt MA (2014). MicroRNAs regulate tight junction proteins and modulate epithelial/endothelial barrier functions. Tissue Barriers.

[58] Tornavaca O, Chia M, Dufton N, Almagro LO, Conway DE, Randi AM, Schwartz MA, Matter K, Balda MS (2015). ZO-1 controls endothelial adherens junctions, cell-cell tension, angiogenesis, and barrier formation. J Cell Biol.

[59] Weksler BB, Subileau EA, Perriere N, Charneau P, Holloway K, Leveque M, Tricoire-Leignel H, Nicotra A, Bourdoulous S, Turowski P, Male DK, Roux F, Greenwood J (2005). Blood-brain barrier-specific properties of a human adult brain endothelial cell line. FASEB J.

[60] Shen H, Wu W (2015). Study of melanin bleaching after immunohistochemistry of melanin-containing tissues. Appl Immunohistochem Mol Morphol.

[61] McCarty KS, Szabo E, Flowers JL, Cox EB, Leight GS, Miller L, Konrath J, Soper JT, Budwit DA, Creasman WT, Seigler HF, McCarty KS (1986). Use of a monoclonal anti-estrogen receptor antibody in the immunohistochemical evaluation of human tumors. Cancer Res.

[62] Hirschhorn T, Barizilay L, Smorodinsky NI, Ehrlich M (2012). Differential regulation of Smad3 and of the type II transforming growth factor-beta receptor in mitosis: implications for signaling. PLoS One.

[63] Thomsen LB, Burkhart A, Moos T (2015). A Triple Culture Model of the Blood-Brain Barrier Using Porcine Brain Endothelial cells, Astrocytes and Pericytes. PLoS One.

[64] Zubrilov I, Sagi-Assif O, Izraely S, Meshel T, Ben-Menahem S, Ginat R, Pasmanik-Chor M, Nahmias C, Couraud PO, Hoon DS, Witz IP (2015). Vemurafenib resistance selects for highly malignant brain and lung-metastasizing melanoma cells. Cancer Lett.

[65] Ozato K, Sachs DH (1981). Monoclonal antibodies to mouse MHC antigens. III. Hybridoma antibodies reacting to antigens of the H-2b haplotype reveal genetic control of isotype expression. J Immunol.

[66] Tomayko MM, Reynolds CP (1989). Determination of subcutaneous tumor size in athymic (nude) mice. Cancer Chemother Pharmacol.

